# Functional outcomes in the Cleft Care UK study – Part 3: oral health and audiology

**DOI:** 10.1111/ocr.12110

**Published:** 2015-11-16

**Authors:** J. Smallridge, A. J. Hall, R. Chorbachi, V. Parfect, M. Persson, A. J. Ireland, A. K. Wills, A. R. Ness, J. R. Sandy

**Affiliations:** ^1^ South Thames’ Cleft UnitGuy's and St Thomas HospitalLondonUK; ^2^ Cleft Net East Cleft NetworkAddenbrooke's HospitalCambridgeUK; ^3^ Children's Hearing CentreUniversity Hospitals Bristol NHS Foundation TrustBristolUK; ^4^ School of Social and Community MedicineUniversity of BristolBristolUK; ^5^ North Thames Cleft CentreGreat Ormond Street Hospital for Children and Broomfield HospitalLondonUK; ^6^ East of England Cleft NetworkAudiologyCambridge University Hospitals NHS Foundation TrustCambridgeUK; ^7^ Centre for Appearance ResearchUniversity of the West of EnglandBristolUK; ^8^ School of Oral and Dental SciencesUniversity of BristolBristolUK; ^9^ National Institute for Health Research (NIHR) Biomedical Research Unit in Nutrition, Diet and Lifestyle at the University Hospitals Bristol NHS Foundation Trust and the University of BristolBristolUK

**Keywords:** audiology, cleft Lip, cleft palate, oral health, treatment outcome

## Abstract

**Objectives:**

To compare oral health and hearing outcomes from the Clinical Standards Advisory Group (CSAG, 1998) and the Cleft Care UK (CCUK, 2013) studies.

**Setting and sample population:**

Two UK‐based cross‐sectional studies of 5‐year‐olds born with non‐syndromic unilateral cleft lip and palate undertaken 15 years apart. CSAG children were treated in a dispersed model of care with low‐volume operators. CCUK children were treated in a centralized, high volume operator system.

**Materials and methods:**

Oral health data were collected using a standardized proforma. Hearing was assessed using pure tone audiometry and middle ear status by otoscopy and tympanometry. ENT and hearing history were collected from medical notes and parental report.

**Results:**

Oral health was assessed in 264 of 268 children (98.5%). The mean dmft was 2.3, 48% were caries free, and 44.7% had untreated caries. There was no evidence this had changed since the CSAG survey. Oral hygiene was generally good, 96% were enrolled with a dentist. Audiology was assessed in 227 of 268 children (84.7%). Forty‐three per cent of children received at least one set of grommets – a 17.6% reduction compared to CSAG. Abnormal middle ear status was apparent in 50.7% of children. There was no change in hearing levels, but more children with hearing loss were managed with hearing aids.

**Conclusions:**

Outcomes for dental caries and hearing were no better in CCUK than in CSAG, although there was reduced use of grommets and increased use of hearing aids. The service specifications and recommendations should be scrutinized and implemented.

## Introduction

Children born with cleft lip and palate are at risk from oral health issues because teeth adjacent to the cleft may be misshapen, of poor quality or missing altogether, additional loss of teeth as a result of preventable dental caries may further compromise dentition. Children with a cleft have a higher incidence of tooth decay 1, 2, 3, and a recent meta‐analysis of literature for young people with non‐syndromic clefting demonstrated this susceptibility with an increased caries prevalence in both the primary and permanent dentition 4. Risk factors include a higher prevalence of teeth with anomalies of enamel and dentine 5, 6 and a longer clearance time for food from the mouth allowing generation of fermentable sugars from starches 7. In addition remedial dental treatment may also be complicated by the children with clefts having higher anxiety associated with treatment 8. When all types of cleft are considered, additional syndromes are seen in about 30% of the affected children and these may have a further influence on their health 9 that may make dental treatment more complex. The consequences of caries may be to increase the burden of care through additional treatment visits and increased risk of pain and risk of sepsis. Children with cleft lip and palate are more likely to have a general anaesthetic for dental treatment before the age of seven years than their peers 10, and this adds further to their number of hospital admissions.

All of these factors point to a need for dental prevention. There are programmes in Scotland and Wales which have shown it is possible to reduce caries in high‐risk groups of children 11. In the 1998 CSAG study, a major concern was the poor oral health status of all children recruited. There were very high levels of filled teeth and untreated caries in both the five‐year‐olds and twelve‐year‐olds. Not surprisingly, as a result of the high level of dental decay in these children and with much of this disease being left untreated, the CSAG made a recommendation that a paediatric dentist should be part of the cleft multidisciplinary team caring for these children 12.

Middle ear disease and hearing problems are common in infants and children with cleft palate 13, 14; abnormal functioning of the Eustachian tube as a result of abnormalities of the palate muscles at the nasopharynx and the palate may lead to build up of otitis media with effusion (OME) 15. Hearing loss from OME is prevalent in children born with a cleft palate, particularly in the early years although this reduces with age 16. Given that there is a high prevalence of middle ear disease in children with cleft palate, grommets may be fitted at the time of palate closure. In the UK, there are published clinical guidelines for placement of grommets in non‐cleft children 17; there is no defined care pathway for children with cleft palate although the NICE guidelines recommend that grommets should only be inserted at palate closure after careful otological and audiological assessment. Two recent systematic reviews 18, 19 examined whether routine early placement of grommets had any advantage for hearing or speech and language development. There was insufficient evidence on which to base clinical guidelines and a clearly identified need for further studies. The use of hearing aids is an alternative to grommets to manage the hearing loss caused by OME 17. The 1998 CSAG study reported wide variation in ENT/Audiology quality across service providers (scored on patient access to assessment and management, availability of appropriate test facilities, clinical coordination, record keeping and audit). The report recommended that a centralized cleft service should include a coordinating ENT surgeon/audiological physician and children should have regular audiological assessments. There was no specific guidance about management of otitis media or hearing loss 12.

The aim of this report is to describe the functional status of CCUK children in terms of their oral health and ear and hearing status and to compare these outcomes with those reported in the CSAG study.

## Subjects and methods

### Study design and sample

The data were derived from two cross‐sectional studies that took place 15 years apart – the CSAG and CCUK studies. In both studies, we recruited children born with non‐syndromic unilateral cleft lip and palate (UCLP) over a defined period. The total sample size was 268 and 239 in the CCUK and CSAG study, respectively. Details of the recruitment and selection of children into these studies can be found elsewhere 20.

#### Oral health

These data were collected with a proforma which was based on the original CSAG data collection sheet. Data on dental caries were collected using the British Association for the Study of Community Dentistry (BASCD) criteria 21 with caries being diagnosed at the dentinal threshold using visual criteria. All observers were consultants in paediatric dentistry who had attended a calibration training and calibration exercise day prior to the start of the data collection. The calibration included examples of children who had been born with a cleft. Using the decayed, missing, filled teeth (dmft) format, levels of caries and the treatment received for caries were recorded. Dental anomalies of upper incisors were scored using the modified developmental defects in enamel (DDE) index 22, and oral hygiene was recorded as good/fair/poor relating to the amount of plaque found on the surface of a lower incisor, none/less than 1/3rd/greater than 1/3rd. A lower incisor was used, or if absent a lower canine, as being more representative of the general level of oral hygiene than an upper incisor associated with the cleft. The presence or absence of sepsis (pus relating to a dental infection) to visual examination of the gingivae was recorded (yes/no). The attendance pattern at the dentist and type of dentist seen routinely was recorded.

#### Audiology

Pure tone audiometry was conducted in the hospital audiology clinics according to British Society of Audiology [BSA standards] 23. Air, and where required, bone conduction hearing threshold levels were recorded. Masking was conducted when required and if possible. A conductive hearing loss was defined as an air bone gap of at least 15 decibel (dB) at two or more adjacent frequencies with air conduction levels greater than 20 dB. Mean hearing threshold levels were calculated with thresholds at 0.5, 1, 2 and 4 kHz, and hearing was categorized as follows: normal hearing (<= 20 dB), mild hearing loss (21–40 dB), moderate hearing loss (41–70 dB) and severe/profound hearing loss (>70 dB).

The appearance of the tympanic membrane on otoscopy was noted and coded as normal or abnormal, based on Flynn et al. 16. Abnormal was defined as either middle ear effusion, active or inactive perforation of the ear drum, retraction, grommet or T‐tube *in situ* or cholesteatoma.

Tympanometry was used to determine middle ear function, and tympanograms were coded according to Jerger's classification 24. Details of previous grommets or T‐tube placement over the first 5 years of life as well as details of any other ENT surgery were obtained from medical notes and parental report. Information on previous and current hearing aid treatment was also recorded.

### Statistical analysis

Summary statistics are presented as means and standard deviations for continuous variables and percentages for categorical variables. Comparisons of outcomes between CSAG and CCUK were done where data exist in both surveys using confidence intervals and hypothesis tests based on a normal approximation because the sample size was sufficiently large (>400).

## Results

### Oral health

In all, 264 children of 268 (98.5%) had some form of oral health assessment. The median age was 5.5 years (IQR: 5.4, 5.7), and 178 (67.4%) were boys. Table 1 shows the oral health characteristics of children in CCUK and CSAG (where available). The mean dmft was 2.3 (95% CI: 1.9, 2.7) in the CCUK children; there was no evidence of a difference compared to the CSAG study. Forty‐eight per cent of children (95% CI: 42, 54) were caries free, and 45% (95% CI: 39, 51) had untreated caries; again there was no evidence that this had changed since the CSAG survey. Overall 4.2% of the children had sepsis at the time of examination. Oral hygiene levels were generally good with 68.7% of the children having good oral hygiene and only 2.3% poor, having plaque covering more than 1/3rd of the scored teeth.

**Table 1:** Summary of dental health characteristics in CCUK children (n = 264 unless stated) and CSAG children (n = 239) where available – results are frequencies and percentages unless stated

			CCUK‐CSAG	
	CCUK	CSAG	Difference: (95% CI)	*p*‐value
Mean dmft	2.3	2.23	0.12 (−0.45, 0.70)	0.7
Caries free (dmft=0)	126 (47.7%)	108 (45.2%)	2.5% (−6, 11)	0.6
Untreated caries (dt>0)	118 (44.7%)	96 (40.2%)	4.5% (−4.1, 13.2)	0.30
Sepsis	11/259 (4.2%)	–	–	–
Oral hygiene (visible deposits)				
None	173/250 (69.2%)	–	–	–
<1/3 of teeth	72/250 (28.8%)	–		
≥1/3 visible deposits	5/250 (2%)	–		

Table 2 shows the overall distribution of regular dental care providers. Patient reported enrolment at a general dental practice was 96% which was similar to the 95% reported in the CSAG study (difference: 1%; 95% CI: −2.8, 4.8; *p* = 0.6). A small percentage (5.3%) reported that they were receiving dental treatment in a hospital setting. The mean dmft was lower when there was a paediatric dentist working within the cleft team; however, there was no statistical evidence of a difference (mean difference: −0.39; 95% CI: −1.27, 0.49, *p* = 0.38).

**Table 2:** Regular care provider for CCUK children (n = 225)

Regular dental care provider	N (%)
General dental practitioner	181 (80.4%)
Community dental service	22 (9.8%)
Hospital	12 (5.3%)
Other	1 (0.4%)
Not enrolled	9 (4.0%)

### Audiology

A total of 227 of 268 (84.7%) children had some form of audiological assessment. The median age was 5.6 (IQR: 5.4, 5.7), and 151 (66.5%) were boys.

### History of Ear, Nose and Throat (ENT) treatment

Table 3 summarizes the data on grommet and T‐tube operations in CCUK and CSAG where available. The most common ENT intervention was grommet surgery, 43% (98/227) of children received at least one set of grommets. Compared to the CSAG survey (152/250 = 61%), this was a reduction of 17.6% (95% CI: 8.8, 26.4, *p* < 0.001). There was also weak evidence of a decrease in the number of multiple insertions of grommets in the CCUK cohort compared to the CSAG study (34% v 46%; difference: −11.6%, 95% CI: −1, −24.1; *p* = 0.073). The median age of insertion was 2.7 years (IQR: 1.3, 4.1), 3.6 years (IQR:2.6, 4.8) and 2.3 years (IQR: 1, 4.4) for the first, second and third treatments. Of those children with grommets, 23 of 91 (25.3%) had them inserted at the same time as their palate closure. In addition, one child had an operation for cholesteatoma, and one had a nasal operation.

**Table 3:** Summary of grommet and T‐tube operations

	CCUK		CSAG	*p* (for a test of difference)
	No (%) of children		No (%) of children	
Grommets ever inserted		98/227 (43%)	152/250 (61%)	<0.001
No of sets of grommets per child undergoing middle ear ventilation surgery^a^	1	61/93 (66%)	68/152 (45%)	0.048
	2	26/93 (28%)	47/152 (31%)	
	3	6/93 (6.5%)	23/152 (15%)^b^	
T‐tubes ever inserted		3/227 (1.3%)	–	–
Grommets or T‐tubes ever inserted		99/227 (44%)	–	–
No of sets of grommets or T‐tubes per child undergoing middle ear ventilation surgery a	1	59/93 (63.4%)	–	–
	2	28/93 (30.1%)	–	–
	3	6/93 (6.5%)	–	–

a Five of 98 had missing data for information on n of grommets inserted.

b Six of 23 reported here had more than three sets of grommets inserted; the chi‐squared test includes these as separate categories.

### History of hearing aid treatment

Twenty‐four children (10.6%, 95% CI: 6.9, 15.3) had been fitted with hearing aids, with most children receiving two hearing aids at the first fitting (75%; 18/24 children). Air conduction aids (AC) were more commonly fitted than bone conduction (BC) aids (14 AC; 4 BC; 6 missing data). Seven children were fitted with hearing aids on more than one occasion. Of the 24 children fitted with hearing aids, 17 had also received one or more set of grommets. Sixteen of the 24 children fitted with hearing aids were still wearing them at the age 5 assessment and had mean hearing levels (better hearing ear) of 28 dB (SD 14 dB).

### Middle ear status

From the otoscopy results, there were 115 of 227 (50.7%; 95% CI: 44.0, 57.3) children with abnormal middle ear status in one or both ears, defined as either middle ear effusion, perforation, grommet or T‐tube *in situ*, tympanic membrane retraction or cholesteatoma. Table 4 shows the number of cases according to the different abnormalities. Grommets and middle ear effusion were the most common. There were no cases of cholesteatoma.

**Table 4:** No of cases (%) with middle ear abnormalities observed on otoscopy (categories are not mutually exclusive)

Ears affected	Middle ear effusion	Perforated ear drum (active or inactive)	Grommet/T tube *in situ* (patent or blocked)	Tympanic membrane retraction	Cholesteatoma
One ear	24 (10.6%)	5 (2%)	36 (15.9%)	15 (6.6%)	0 (0%)
Both ears	24 (10.6%)	1 (0.4%)	23 (10.1%)	12 (5.3%)	0 (0%)
Either ear	48 (21%)	6 (2.6%)	59 (26%)	27 (12%)	0 (0%)

Tympanometry results were available from at least one ear of 196 children and 379 ears of 454 (Table 5). There were 41 of 227 (18.1%; 95% CI: 13.3%, 23.7%) of children who had normal middle ear function, defined as type A tympanograms, in both ears at age 5; 108 (48%) children had a type B tympanogram in at least one ear and 61 children in both ears (27%).

**Table 5:** Tympanometry results showing the function of the middle ear [results shown by child (%)]

		Left ear			
		Type A	Type B	Type C	Missing
Right ear	Type A	41 (18%)	12 (5%)	16 (7%)	1 (0.4%)
	Type B	9 (4%)	61 (27%)	8 (3.5%)	5 (2.2%)
	Type C	11 (5%)	9 (4%)	16 (7%)	1 (0.4%)
	Missing data	1 (0.4%)	4 (2%)	1 (0.4%)	31 (14%)

Type A: Normal peaked tympanogram (−150 to 50 daPa) indicates normal middle ear function; type B: flat tympanogram indicates reduced compliance of tympanic membrane; type C: tympanogram with negative middle ear pressure <−150 daPa.

### Hearing threshold levels

**Figure 1:**
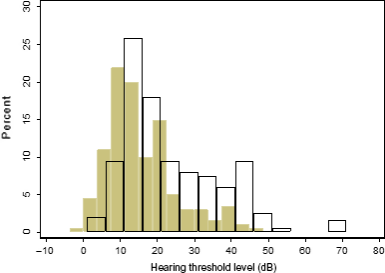
Distribution of mean air conduction hearing thresholds averaged 0.5–4 kHz (dB) according to best (closed bars) and worst ear (open bars) in CCUK. Normal hearing equates to hearing thresholds of 20 dB or less.

Hearing threshold data were available from at least one ear of 201 children (Figure 1). The percentage with mild or greater levels of hearing loss in the better hearing ear was 22%, of these 1.5% had a moderate or greater hearing loss. There was no evidence this was different compared to CSAG, where the figures were 19% and 2%, respectively (*p* = 0.8). In the worst hearing ear, 44% of children had a mild or greater hearing loss; 14% had a moderate or greater loss. Overall 56% had normal hearing, 22% had a unilateral hearing loss, and 22% had a bilateral hearing loss. The hearing losses were primarily conductive with only one bilateral sensorineural case and two cases of bilateral mixed hearing losses (prevalence of bilateral sensorineural hearing loss: 1.3%; 95% CI: 0.2%, 3.8%). In addition, five unilateral cases did not have enough test information to determine the type of hearing loss.

## Discussion

This analysis of outcomes for children born with cleft lip and palate had two main aims in examining dental caries and hearing in two cross‐sectional studies of children born with a cleft lip and palate 15 years after a centralized model of care was introduced in the UK. There were some disappointing results. First, there was no difference in dmft between CCUK and CSAG; second, the prevalence of untreated caries remained the same despite the fact that, within the CCUK cohort, oral hygiene, generally was good in most of the young people and that nearly all were registered with a dentist. Not all multidisciplinary teams (MDTs) had a paediatric dentist attached, and although there have been some improvements in the number of five‐year‐old children with a cleft remaining caries free, the results are still disappointing. It is worth documenting the intentions of centralization in the original CSAG study. Unlike most other aspects of the CCUK study, the recommendations for dental care have been interpreted differently by different units around the country. The minimum standards for the management of oral health in children born with cleft lip and palate recommended that dental health education should be the responsibility of a named member of the team. The cleft team should ensure that dental health education, fluoride supplementation and dental attendance are maintained throughout childhood. To ensure this is coordinated, children with repaired clefts should have priority access to a consultant in paediatric dentistry where necessary. If this consultant is not a team member, access should be available for treatment planning at an early enough stage to be able to influence outcome. Unfortunately, at the time of the CCUK study, only five of the 11 regional cleft units had managed to persuade commissioners that a consultant in paediatric dentistry needed to be part of the MDT. All of these were employed on a part time basis, and only in three regions did the consultant in paediatric dentistry have a regular presence at the MDT clinics. At the time when these children were born and first dental prevention should have been done, the figures were even less. Therefore, many of the children in this study would not have been seen previously by a paediatric dentist. Non‐dental members of the team screening for oral health would have only been able to detect overt dental disease and would not have been able to carry out a caries risk assessment to identify children in the higher risk categories. The fact that the average dmft had shown no change compared to the CSAG study level 25 suggests the lack of consistent input from paediatric dentists is still an aspect of UK cleft provision that needs to be improved. The percentage of children who were caries free had remained similar from 45% to 48%, this is below the BASCD reported caries free percentages which has improved from 64.3% (1999) to 72.1% (2012). There needs to be caution when interpreting these figures. The 1999 and 2012 BASCD studies are not directly comparable because the permissions for inclusion of subjects changed to a positive consent process (rather than an opt‐out) in the 2007–2008 survey. The impact of seeking this positive consent appears to have depressed the caries severity and prevalence reported in Wales and England 26.

There is evidence that the teeth around the site of the cleft have a higher prevalence of enamel discolouration 27, with 56% of four‐year‐olds having at least one incisor enamel opacity 3. Enamel hypoplasia, defined as a quantitative disturbance of mineralized tissue formation during tooth development, may leave a tooth particularly vulnerable to dental caries 28. Two other studies have found that the highest prevalence of caries is in the teeth around the cleft particularly in lateral incisors 29, 30. Caries prevalence in specific teeth will be reported in further papers, but it is difficult to study this aspect in five‐year‐old children. The standardized scoring convention in calibrated studies is that children with missing incisors at 5 years old are considered to have lost the teeth through exfoliation not extraction. Therefore, early loss of incisors through caries is not recorded in the dmft of five‐year‐olds. Assessment of children with clefts at 5 years of age is too late to identify these risk factors. In two‐year‐old children born with a cleft in Taiwan, 15.4% had rampant caries (baby bottle tooth decay) and needed extensive dental treatment to restore oral health 31. The lack of reduction in caries found in CCUK may reflect the fact that in the majority of centres, children are not receiving adequate assessment of their dental needs at an early enough date and are not receiving the intense prevention needed to reduce caries levels. Population wide caries prevention programmes such as Childsmile (Scotland) 11 and Designed to smile (Wales) 32, have shown that intensive prevention programmes started early enough, and which identify high caries risk children can give dramatic improvements in oral health. However, these schemes are labour intensive and need to be adequately funded.

The lack of regular screening and use of simple preventive procedures such as fluoride varnish applications will also be reported in more detail in a subsequent publication. In the CLAPA report on users’ perspectives of cleft care only 18% of families had met a paediatric dentist 33, 96% of the children in CCUK reported being enrolled with a dental practitioner and yet in 33.8% of these children no attempt had been made to treat the dental disease present. This along with the national figures in the BASCD studies is a sad reflection on the standard of dental care offered to children in the UK. Untreated dental disease can lead to pain and infection which has an impact on the quality of life 34. In a recent survey of five‐year‐old children in Glasgow, 28.7% reported difficulty eating, 18.5% difficulty sleeping and 14.9% avoided smiling due to toothache 35, with 4.2% of the children having dental infections present in their mouth at the time of examination. The service specification for cleft care highlights the need for dental prevention and care within each cleft service, and this should result in improved oral health if followed and fully implemented.

With regard to hearing, there was evidence (albeit weak) of a decrease in the number of multiple insertions of grommets in the CCUK cohort compared to the CSAG study. Furthermore, grommet surgery episodes were reduced and the number of multiple insertions decreased. In an Australian study of cleft care 36 69% of five‐year‐olds had grommets inserted compared with 61% of UK five‐year‐olds in the CSAG study and 43% in the CCUK. In the current study, we found that 10% of children had grommets inserted simultaneously with palatal closure. There is as yet insufficient evidence to guide best practice for grommet treatment in children with cleft palate 18, 19. Some surgical teams, for example Andrews et al. and Shaw et al. take a cautious approach to early grommet insertion 37, 38, whereas others, for example Merrick et al., perform simultaneous grommets and palatal closure for all children 39. The results described indicate that most cleft centres in the UK are taking a cautious approach, with over 75% of grommets inserted conservatively.

Most children had normal average hearing levels in one or both ears but about 20% had a bilateral hearing loss which was primarily conductive. It is not possible from these cross‐sectional data to determine the persistence of the hearing losses, although a quarter of cases were being managed by hearing aids or had grommets *in situ*. The proportion with hearing loss has not changed since the CSAG study, possibly because approaches to management have altered with hearing aids now being used (in the 1998 CSAG, there were no reports of hearing aid treatment being offered). This is important as it is how hearing loss is managed rather than the hearing loss itself which determines the impact that hearing difficulties may have on listening, language and other aspects of development. Hearing aids are now being offered as a treatment option with around 10% being treated with these and often before or after grommet surgery. The latter is a predictor of hearing aid treatment implying that hearing aids are being offered as an alternative option to multiple sets grommets.

Fifty‐one per cent of children had abnormal middle ear status in one or both ears. The equivalent data were not available from CSAG for comparison so it is not possible to determine whether there has been any improvement. It would be expected that the introduction of regular audiological assessments post‐CSAG would lead to a reduction in cases of middle ear disease as a result of earlier detection. Flynn et al. 16, who used similar criteria and a sample of five‐year‐olds with UCLP in Sweden, found 55% of ears had abnormal middle ear status. Middle ear abnormalities are still common in this cohort but have been shown to reduce with age 16. Children with cleft palate are at increased risk of cholesteatoma 40, and prevalence has been reported to be between 1.8 and 5.9% 41. There were no cases in CCUK presenting with cholesteatoma at age 5, and only one case had received prior treatment for cholesteatoma indicating a low incidence in this cohort at this age. It will be important to continue to follow‐up the CCUK cohort to determine whether middle ear abnormalities and the incidence of cholesteatoma decrease or increase with age.

The decrease in grommet use may be related to the changes in cleft care delivery or the introduction of the NICE guidelines 17. Hearing aid provision is now considered a suitable option to manage hearing impairment. There were no specific ENT/audiology recommendations in CSAG (1998) although the current clinical standards for ENT and audiology care identify the minimum set of hearing assessments and the requirement for hearing loss to be managed 42. Changes in measurement methodology and acceptance thresholds may account for these differences, but there is no good evidence to inform the management of hearing difficulties in children born with a cleft palate.

ENT and audiology care is the joint responsibility of the central and local teams within the Cleft network. There is variation in the way assessments, and interventions are conducted both within and across networks. This may be due to practicalities and logistics of delivering the service rather than a result of clinical need. Local audiology teams may also be unaware of the care standards for cleft palate which do not have the same high profile as, for example the audiology care standards for Down Syndrome 43, a group of children who also have increased risk of OME and hearing loss. The lines of accountability across the central and local teams within each network should therefore be clearly defined. Given the primary cleft surgery and preschool age, interventions are implemented in the central hubs, and decisions relating to management of early hearing loss may be more optimally managed centrally where the communication channels across professionals are likely to be more robust. The ENT/Hearing Special Interest Group of the Craniofacial Society of Great Britain and Ireland is working to address some of these issues and provides a national forum to present audit results. A set of cleft specific outcome measures are currently being developed to evaluate audiological treatment and management for children with cleft palate 44, and these could be useful for auditing audiological care and management across centres. Further research is needed to establish the effectiveness of interventions to treat hearing impairment in children with cleft of all ages.

## Conclusions

Overall the centralization of cleft services in the UK has had little impact on oral health of children born with UCLP. The most pressing issue is to implement fully the recommendations made following CSAG with regard to provision of dental care and service. Outcomes for hearing were no better in CCUK than in CSAG, although there was reduced use of grommets and increased use of hearing aids. These two aspects of cleft care in the UK would benefit from further scrutiny of service specifications and support.

## Clinical relevance

Two key outcomes in children with a cleft are oral health and ability to hear. The latter function will also impact on speech development, and together, they may affect well‐being and development. In the 1998 Clinical Standards Advisory Group study, oral health outcomes were poor and it was hoped that the centralization of cleft services in the UK (a reduction of cleft centres from 57 to 11) would improve this. The implementation of paediatric dental services and ENT/audiology into centralized multidisciplinary care has been slow and incomplete and has yet to have significant impact on oral health and hearing.
